# *Notes from the Field:* Carbapenemase-Producing Carbapenem-Resistant Enterobacteriaceae from Less Common Enterobacteriaceae Genera — United States, 2014–2017

**DOI:** 10.15585/mmwr.mm6723a4

**Published:** 2018-06-15

**Authors:** Maroya S. Walters, Medora Witwer, Yeon-Kyeng Lee, Valerie Albrecht, David Lonsway, J. Kamile Rasheed, Melissa Anacker, Paula Snippes-Vagnone, Ruth Lynfield, Alexander J. Kallen

**Affiliations:** ^1^Division of Healthcare Quality Promotion, National Center for Emerging and Zoonotic Infectious Diseases, CDC; ^2^Minnesota Department of Health; ^3^CDC Korea, South Korea.

Infections with carbapenemase-producing carbapenem-resistant Enterobacteriaceae (CP-CRE) are associated with high mortality rates (*1*). Carbapenemases encoded on plasmids can move between bacterial strains and have the potential to rapidly increase the proportion of Enterobacteriaceae resistant to carbapenems; as such, CP-CRE have been a particular focus of public health response. Although the Enterobacteriaceae family includes approximately 50 recognized genera, surveillance for CP-CRE has focused on the organisms most associated with clinical infections: *Klebsiella* spp., *Enterobacter* spp., and *Escherichia coli* (*2*,*3*). CRE from other, less commonly encountered genera (hereafter referred to as less common genera) have generally not been targeted for carbapenemase testing, in part, because some of these organisms possess intrinsic resistance to the carbapenem imipenem and others express species-specific chromosomal carbapenemases. However, these organisms can also harbor plasmid-mediated carbapenemases. This report describes CP-CRE from less common genera identified through reference testing at CDC and surveillance at the Minnesota Department of Health (MDH) Public Health Laboratory.

CDC’s Clinical and Environmental Microbiology Branch performs molecular testing on submitted CRE to detect *Klebsiella pneumoniae* carbapenemase (KPC), New Delhi metallo-β-lactamase (NDM), Verona integron-mediated metallo-β-lactamase (VIM), active-on-imipenem (IMP), and OXA-48-type carbapenemases. During January 1, 2014–May 25, 2017, CDC identified 1,039 CP-CRE, including 63 (6.1%) from the less common genera. Isolates from the less common genera were submitted by 23 states; Iowa (10; 16%) and Pennsylvania (seven; 11%) contributed the most. KPC-producing *Citrobacter* spp. (27; 42.9%) was the most common organism-mechanism combination identified (Table).

**TABLE:** Carbapenemase-producing carbapenem-resistant Enterobacteriaceae by species and mechanism, among organisms other than *Klebsiella spp*., *Enterobacter spp*., and *E. coli* tested at CDC, January 1, 2014–May 25, 2017, and the Minnesota Department of Health (MDH) Public Health Laboratory, January 1, 2014–September 10, 2017*

Laboratory (period)/Organism	Mechanism					Total
	KPC	IMP	NDM	OXA-48 type	VIM	
**CDC (January 1, 2014–May 25, 2017)^†^**						
***Citrobacter* ^§^**	27	0	1	0	1	**29**
*Citrobacter freundii*	22	0	1	0	1	**24**
*Citrobacter koseri*	2	0	0	0	0	**2**
*Citrobacter braakii*	1	0	0	0	0	**1**
*Citrobacter farmeri*	1	0	0	0	0	**1**
***Morganella* ^§^**	0	1	2	0	0	**3**
*Morganella morganii*	0	1	1	0	0	**2**
***Proteus mirabilis***	6	2	2	0	1	**11**
***Providencia* ^§^**	1	8	1	0	0	**10**
*Providencia rettgeri*	0	6	0	0	0	**6**
*Providencia stuartii*	1	1	1	0	0	**3**
***Raoultella* ^§^**	4	0	0	0	1	**5**
*Raoultella ornithinolytica*	1	0	0	0	0	**1**
***Serratia***	4	0	0	0	1	**5**
*Serratia marcescens*	3	0	0	0	1	**4**
*Serratia ureilytica*	1	0	0	0	0	**1**
**Total, CDC**	**42**	**11**	**6**	**0**	**4**	**63**
**MDH Public Health Laboratory (January 1, 2014–September 10, 2017)**						
*Citrobacter freundii*	6	0	1	0	0	**7**
*Providencia rettgeri*	0	7	1	0	0	**8**
*Serratia marcescens*	1	0	0	0	1	**2**
*Raoultella planticola*	1	0	0	0	0	**1**
*Raoultella ornithinolytica*	0	0	0	1	0	**1**
*Leclercia adecarboxylata*	1	0	0	0	0	**1**
**Total, MDH Public Health Laboratory**	**9**	**7**	**2**	**1**	**1**	**20**

**Abbreviations:** CP-CRE = carbapenemase-producing carbapenem-resistant Enterobacteriaceae; IMP = active-on-imipenem; KPC = *Klebsiella pneumoniae* carbapenemase; NDM = New Delhi metallo-β-lactamase; VIM = Verona integron-mediated metallo-β-lactamase.

* Two IMP-producing *Providencia rettgeri* and one VIM-producing *Serratia marcescens* from Minnesota are included in both the CDC and MDH Public Health Laboratory sections of the table.

^†^ Limited to isolates submitted to CDC for confirmatory testing; not all passively reported CP-CRE patients had isolates submitted.

**^§^** One KPC-producing *Citrobacter*, one NDM-producing *Morganella*, one IMP-producing *Providencia*, three KPC-producing *Raoultella,* and one VIM-producing *Raoultella* were not identified to species level.

CRE producing a carbapenemase other than KPC are historically uncommon in the United States and often associated with health care exposures outside the United States. Epidemiologic data were available for 20 of 28 patients with non–KPC–CP-CRE from less common genera passively reported to CDC during this period. The median patient age was 62.5 years (range = 2 months to 79 years). Travel history in the year preceding the positive culture was reported for 18 patients; 10 did not travel outside the United States, including five from a single cluster. Six patients were hospitalized outside the United States: three in India, and one each in the Philippines, Romania, and Spain.

The Minnesota Department of Health initiated surveillance for all CRE species in Hennepin and Ramsey counties in 2012 and expanded surveillance statewide on January 1, 2016. Isolates submitted to the MDH Public Health Laboratory are tested for carbapenemases. During January 1, 2014–September 30, 2017, 149 (12%) of 1,241 CRE submitted were carbapenemase-producing; the percentage did not differ between isolates from the more common (*Klebsiella spp.*, *Enterobacter spp.*, and *E. coli*) and the less common genera. Among the 149 CP-CRE isolates, all were from unique patients, and 20 (13.4%) were from less common genera. The most common organism and mechanism combinations among the less common genera were IMP-producing *Providencia rettgeri* (seven; 35%) and KPC-producing *Citrobacter freundii* (six; 30%) (Table).

Epidemiologic data were available for the 20 Minnesota patients with CP-CRE from the less common genera; no clusters were identified. The median patient age was 56.5 years (range = 14–75 years), and 15 (75%) patients were hospitalized at the time of culture collection. Two patients were hospitalized internationally (one each in Kenya and Kuwait) in the year before their positive culture.

Less common Enterobacteriaceae genera appear to be a small but potentially important subset of CP-CRE; however, estimates of the true proportion of CP-CRE from these less common genera are limited by the lack of systematic testing. Of note, many of the carbapenemases in the less common CRE genera were not KPC. These were frequently identified in patients who did not report travel outside the United States in the year preceding their positive culture, indicating domestic acquisition. Clinicians should be aware that CRE from the less common genera can harbor carbapenemases and consider requesting carbapenemase testing from state public health laboratories to guide infection control practices and prevent further spread of these resistance mechanisms. CRE surveillance that includes a broader range of Enterobacteriaceae genera is being piloted in 10 states and will be critical for better understanding the potential impact of these less common genera on the spread of carbapenemases.

## References

[1] Tamma PD, Goodman KE, Harris AD, Comparing the outcomes of patients with carbapenemase-producing and non-carbapenemase-producing carbapenem-resistant Enterobacteriaceae bacteremia. Clin Infect Dis 2017;64:257–64. 10.1093/cid/ciw741 28013264 PMC5241781

[2] Guh AY, Bulens SN, Mu Y, Epidemiology of carbapenem-resistant Enterobacteriaceae in 7 US Communities, 2012–2013. JAMA 2015;314:1479–87. 10.1001/jama.2015.12480 26436831 PMC6492240

[3] Weiner LM, Webb AK, Limbago B, Antimicrobial-resistant pathogens associated with healthcare-associated infections: summary of data reported to the National Healthcare Safety Network at the Centers for Disease Control and Prevention, 2011–2014. Infect Control Hosp Epidemiol 2016;37:1288–301. 10.1017/ice.2016.174 27573805 PMC6857725

